# Construction of an XGBoost-SHAP-based malignant transformation risk prediction model for gallbladder polyps

**DOI:** 10.1080/07853890.2026.2659395

**Published:** 2026-04-24

**Authors:** Wen-Hui Luo, Meng-Han Cai, Yu Wang, Ying-Jun Wu, Jun-Fan Yang, Shao-Jun Li

**Affiliations:** ^a^ The Second Department of Hepatobiliary Surgery, Yantai Yuhuangding Hospital, Yantai, Shandong Province, China; ^b^ Department of Dermatology, Affiliated Hospital of Tianjin Academy of Traditional Chinese Medicine, Tianjin, China; ^c^ School of Clinical Medicine, Jining Medical University, Jining, Shandong Province, China; ^d^ Department of Biliary Tract Surgery, The Third Affiliated Hospital of Naval Medical University, Jiading, Shanghai, China; ^e^ Ultrasound Department of the Affiliated Hospital of Shandong College of Traditional Chinese Medicine, Yantai, Shandong Province, China; ^f^ Department of General Surgery, Yantai Yuhuangding Hospital, Yantai, Shandong Province, China

**Keywords:** Gallbladder polyp, malignant transformation, neoplastic polyp, risk prediction model, SHAP, XGBoost

## Abstract

**Background:**

To develop and validate a risk prediction model for malignant transformation in patients with gallbladder polyps (GBPs) using an interpretable machine learning framework and evaluate its predictive accuracy.

**Methods:**

A retrospective cohort of 1,027 surgical patients was enrolled from Yantai Yuhuangding Hospital (training set: *n* = 933) and Shanghai Eastern Hepatobiliary Surgery Hospital (validation set: *n* = 94). Feature selection for the training set was performed using the least absolute shrinkage and selection operator (LASSO) regression method. A predictive model was constructed with the XGBoost machine learning algorithm and evaluated using Shapley Additive exPlanation (SHAP).

**Results:**

LASSO regression identified five significant risk factors for malignant transformation in GBPs: presence of concomitant cholecystitis, polyp count, polyp base width, age, and maximum polyp diameter. The area under the receiver operating characteristic curve (AUC) was 0.862 (95% confidence interval [CI]: 0.8342–0.8893) in the training set and 0.777 (95% CI: 0.6804–0.8737) in the validation set. SHAP analysis illustrated the contribution of each factor.

**Conclusion:**

This study developed and validated a risk prediction model for malignant transformation in patients with GBPs. The model demonstrated favorable discrimination, calibration, accuracy, and clinical applicability. Integration with SHAP technology may assist clinicians in optimizing treatment and management strategies.

## 1. Introduction

### 1.1. Clinical background

Gallbladder polyps (GBPs) are protruding mucosal growths within the gallbladder. The detection rate of GBP by abdominal ultrasound was 4–9% in the general population [1,2]. Most studies categorize GBPs as non-neoplastic polyps (e.g. cholesterol or inflammatory polyps), which constitute over 70% of cases. These lesions are typically asymptomatic and have an extremely low rate of malignant transformation. In contrast, neoplastic polyps such as adenomas and adenocarcinomas, though less common, carry greater clinical significance [3,4]. The malignant transformation rate of adenomas and precancerous lesions (dysplasia and low-grade intraepithelial neoplasia) ranges from 3% to 23.5% [5,6]. The 5-year survival rate for early-stage gallbladder cancer is 75%, while that for advanced-stage disease falls below 5%. However, early-stage gallbladder cancer is extremely difficult to diagnose and is often discovered only incidentally during pathological examination after cholecystectomy [7].

Current clinical guidelines for GBPs primarily recommend surgery for polyps ≥10 mm in diameter. According to European guidelines, 70% of resected polyps are benign lesions [6]. This leads to unnecessary surgical risks and financial burdens for patients. However, 6–9% of polyps <10 mm are histologically confirmed to be adenomas or early-stage cancers [8,9]. The coexistence of overtreatment and undertreatment compromises healthcare delivery.

### 1.2. Research rationale

Multiple international consensus documents, including guidelines from the Japanese Society for Hepato-Biliary-Pancreatic Surgery and the European Society of Gastrointestinal and Abdominal Radiology (ESGAR), advocate for multifactorial malignant transformation risk assessment of polyps [6,10]. This study aims to establish such a preoperative prediction model to optimize surgical decision-making, enhance the detection rate of malignant polyps, reduce unnecessary surgeries, and lower healthcare costs.

## 2. Methods

### 2.1. Data sources

A retrospective cohort study was conducted using data from 1,027 patients who underwent surgery. The training set comprised 933 patients from Yantai Yuhuangding Hospital (2017–2024), and the validation set included 94 patients from Shanghai Eastern Hepatobiliary Surgery Hospital (2023–2024). The cohort included 731 non-neoplastic polyps (cholesterol polyps, inflammatory polyps, and hyperplastic polyps) and 296 neoplastic polyps (adenomas, polyps with low-grade intraepithelial neoplasia, polyps with dysplastic hyperplasia, and adenomyomatosis). Consistent with the classification by Lee and Wiles et al. [5,6], lesions exhibiting atypical hyperplasia and low-grade intraepithelial neoplasia were included in the neoplastic polyp group. Data are summarized in Table 1. The inclusion criteria were: (i) Post-cholecystectomy pathological diagnosis of GBP or gallbladder adenoma; (ii) Complete clinical baseline data. The exclusion criteria were: (i) Acute cholecystitis; (ii) Gallbladder stones or intrahepatic bile duct stones; (iii) IgG4-related sclerosing cholangitis; (iv) A preoperative diagnosis of gallbladder cancer; (v) Concurrent obstructive jaundice.

**Table 1.** Baseline data comparison between training and validation sets.

Variable	Total cases (*n* = 1027)	Cases from Yantai Yuhuangding Hospital (training set: *n* = 933)	Cases from Shanghai Eastern Hepatobiliary Surgery Hospital (validation set: *n* = 94)	*P*
**Sex (%)**				
Male	372 (36)	335 (36)	37 (38)	
Female	655 (64)	598 (64)	57 (62)	
**Clinical diagnosis (%)**				< 0.001
Non-neoplastic polyps	731 (71)	687 (74)	44 (47)	
Neoplastic polyps	296 (29)	246 (26)	50 (53)	
**Concurrent cholecystitis (%)**				< 0.001
Yes	111 (11)	60 (6)	51 (54)	
None	916 (89)	873 (94)	43 (46)	
**Gallbladder stones (%)**				0.078
None	839 (82)	769 (82)	70 (74)	
Yes	188 (18)	164 (18)	24 (26)	
**Gallbladder polyp count (%)**				0.037
Single	493 (48)	458 (49)	35 (37)	
Multiple	534 (52)	475 (51)	59 (63)	
**Polyp formation time (%)**				< 0.001
<1 month	284 (28)	256 (27)	28 (30)	
1–12 months	309 (30)	265 (28)	44 (47)	
12–60 months	275 (27)	262 (28)	13 (14)	
>60 months	159 (15)	150 (16)	9 (10)	
**Polyp echo (%)**				0.563
Hypoechoic	111 (11)	103 (11)	8 (9)	
Weak echo	916 (89)	830 (89)	86 (91)	
**Abdominal symptoms (%)**				0.002
None	812 (79)	750 (80)	62 (66)	
Yes	215 (21)	183 (20)	32 (34)	
**Family history of gallbladder cancer (%)**				0.612
None	1017 (99)	923 (99)	94 (100)	
Yes	10 (1)	10 (1)	0 (0)	
**Smoking history (%)**				0.015
None	944 (92)	851 (91)	93 (99)	
Yes	83 (8)	82 (9)	1 (1)	
**Alcohol consumption history (%)**				0.023
None	950 (93)	857 (92)	93 (99)	
Yes	77 (7)	76 (8)	1 (1)	
**Polyps base (%)**				0.229
Narrow base	622 (61)	571 (61)	51 (54)	
Wide base	405 (39)	362 (39)	43 (46)	
Age, M (Q1, *Q*3)	51 (40, 59)	51 (40, 59)	52.5 (39.25, 60)	0.703
Gallbladder wall thickness, *M* (*Q*1, *Q*3)	2.5 (2, 3)	2.5 (2, 3)	4 (3, 4)	< 0.001
Maximum polyp diameter, *M* (*Q*1, *Q*3)	11.7 (10, 14)	11.7 (10, 14)	12 (10, 17)	0.467
Polyps enlarged, *M* (*Q*1, *Q*3)	2 (0, 5)	2 (0, 5)	1 (0, 3)	0.04
CA_199, *M* (*Q*1, *Q*3)	8.48 (5.5, 13.69)	8.52(5.49,13.38)	8.11(5.59,15.07)	0.791
CEA, *M* (*Q*1, *Q*3)	1.51 (1.05, 2.17)	1.51 (1.05, 2.19)	1.55 (1.07, 2.1)	0.545

### 2.2. Variable screening

To ensure data accuracy and completeness, a double-entry method with cross-verification was employed. The following 18 variables were collected: age, sex (0 = female, 1 = male), abdominal symptoms (0 = no, 1 = yes), alcohol consumption history (0 = no, 1 = yes), polyp formation duration (0 = < 1 month, 1 = 1–12 months, 2 = 12–60 months, 3 = > 60 months), history of gallbladder cancer (0 = no, 1 = yes), polyp enlargement diameter, smoking history (0 = no, 1 = yes), polyp echogenicity (0 = non-hypoechoic, 1 = hypoechoic), polyp base (0 = narrow, 1 = wide), gallbladder wall thickness, maximum polyp diameter (0 ≤ 9 mm, 1 = 10–12 mm, 2 > 12 mm), polyp number (0 = single, 1 = multiple), concurrent cholecystitis (0 = no, 1 = yes), concurrent gallbladder stones (0 = no, 1 = yes), polyp nature, carbohydrate antigen-199, and carcinoembryonic antigen.

### 2.3. Statistical analysis methods

This study employed R 4.5.1 for statistical analysis. Variables were selected using LASSO regression on the training set, and XGBoost was used to construct the predictive model. Clinical efficacy was evaluated using the area under the receiver operating characteristic (ROC) curve (AUC) and accuracy, supplemented by decision curve analysis (DCA). Variable contributions and personalized predictions were evaluated using the Shapley Additive exPlanation (SHAP) analysis. Data cleaning and visualization were performed using the tidyverse, pROC, CBCgrps, and rms packages. *p* < 0.05 was considered statistically significant.

The XGBoost model was constructed using the ‘XGBoost’ package. The ‘train’ function was used to optimize parameters from the input package and output the optimal parameter configuration. The model was established by setting the learning rate (eta) to 0.1 and the maximum depth (max_depth) to 2. Tenfold cross-validation was employed to mitigate the risk of overfitting. The number of iterations (boosting rounds) was set to 100. Model performance was evaluated using ROC curves, calibration curves, and DCA. Predicted probabilities were converted to binary outcomes using a threshold of 0.5. Accuracy, sensitivity, specificity, and other metrics were calculated for both the training and validation sets.

SHAP values were used to explain the XGBoost model. The ‘Shapviz’ is an R package used for interpreting the predictions of a machine learning model. This package provides visual explanations based on SHAP values and quantifies the contribution of each feature (positive or negative) to the model prediction. Feature importance plots display the most influential features, with feature importance ranked by the average absolute value of SHAP values.

## 3. Results

### 3.1. Baseline characteristics of patients with GBPs

Of the 1,027 patients, 296 (29%) had neoplastic polyps and 731 (71%) had non-neoplastic polyps. Among the entire study population, 36% were male, and the median age was 51 years (range: 40–59). Compared with the validation set, the training set had significantly higher proportions of non-neoplastic polyps, solitary GBPs, polyps present for >12 months (12–60 months, >60 months), absence of abdominal symptoms, smokers, alcohol consumers, and cases with polyp enlargement (*p* < 0.05). Conversely, the training set had lower proportions of neoplastic polyps, concomitant cholecystitis, multiple GBPs, polyp formation time of ≤12 months (1–12 months and <1 month), abdominal symptoms, and gallbladder wall thickness (*p* < 0.05, Table 1). No statistically significant differences were observed in gallbladder stones, polyp echogenicity, family history of gallbladder cancer, polyp base, maximum polyp diameter, CA_199, or CEA between the training and validation sets (*p* > 0.05).

### 3.2. Results of training and validation sets

Our study compared the characteristics of 17 indicators between the training and validation sets (Tables 2 and 3). Effective risk factors were subsequently identified within the training set (Table 4). Solitary polyps, polyps exceeding 13 mm in diameter, polyps with a broad base, concomitant cholecystitis, and age ≥55 years were significantly associated with the development of neoplastic polyps, requiring attention from surgeons.

**Table 2.** Data from Yantai Yuhuangding Hospital (training set).

Variable	Total (*n* = 933)	Non-tumor group (*n* = 687)	Tumor group (*n* = 246)	*P*
**Sex (%)**				0.155
Female	598 (64)	450 (66)	148 (60)	
Male	335 (36)	237 (34)	98 (40)	
**Concurrent cholecystitis (%)**				< 0.001
Yes	60 (6)	19 (3)	41 (17)	
None	873 (94)	668 (97)	205 (83)	
Gallbladder stones (%)				0.96
None	769 (82)	567 (83)	202 (82)	
Yes	164 (18)	120 (17)	44 (18)	
**Gallbladder polyp count (%)**				< 0.001
Single	458 (49)	293 (43)	165 (67)	
Multiple	475 (51)	394 (57)	81 (33)	
**Time to polyp formation (%)**				0.078
< 1 month	256 (27)	181 (26)	75 (30)	
1–12 months	265 (28)	198 (29)	67 (27)	
12–60 months	262 (28)	186 (27)	76 (31)	
> 60 months	150 (16)	122 (18)	28 (11)	
**Polyp echoes (%)**				< 0.001
Hypoechoic	103 (11)	56 (8)	47 (19)	
Hypoechoic	830 (89)	631 (92)	199 (81)	
**Abdominal symptoms (%)**				0.743
None	750 (80)	550 (80)	200 (81)	
Yes	183 (20)	137 (20)	46 (19)	
**Family history of gallbladder cancer (%)**				0.14
None	923 (99)	682 (99)	241 (98)	
Yes	10 (1)	5 (1)	5 (2)	
**Smoking history (%)**				1
None	851 (91)	627 (91)	224 (91)	
Yes	82 (9)	60 (9)	22 (9)	
**Drinking history (%)**				0.691
None	857 (92)	633 (92)	224 (91)	
Yes	76 (8)	54 (8)	22 (9)	
**Polyps base (%)**				< 0.001
Narrow base	571 (61)	503 (73)	68 (28)	
Wide base	362 (39)	184 (27)	178 (72)	
Age, *M* (*Q*1, *Q*3)	51 (40,59)	49 (39, 57)	55.5 (47, 64)	< 0.001
Gallbladder wall thickness, *M* (*Q*1, *Q*3)	2.5 (2, 3)	2.5 (2, 3)	2.5 (2, 3.5)	< 0.001
Maximum polyp diameter, *M* (*Q*1, *Q*3)	11.7 (10, 14)	11 (10, 13)	13 (11, 17)	< 0.001
Polyps enlarged, *M* (*Q*1, *Q*3)	2 (0, 5)	2 (0, 5)	2 (0, 6)	0.029
CA_199, *M* (*Q*1, *Q*3)	8.52 (5.49, 13.38)	7.9(5.26, 12.25)	10.2(6.47,16.0)	< 0.001
CEA, *M* (*Q*1, *Q*3)	1.51 (1.05, 2.19)	1.49 (1.02, 2.19)	1.58 (1.13, 2.2)	0.128

As shown in Table 2, gallbladder inflammation, hyperechoic appearance, polyp with a broad base, age, polyp size, and elevated CA-199 levels were high-risk factors in the tumor group.

**Table 3.** Data from Shanghai Eastern hepatobiliary surgery hospital (validation set).

Variable	Total (*n* = 94)	Non-tumor group (*n* = 44)	Tumor group (*n* = 50)	*P*
**Sex (%)**				0.356
Female	57 (61)	24 (55)	33 (66)	
Male	37 (39)	20 (45)	17 (34)	
**Concurrent cholecystitis (%)**				0.795
Yes	51 (54)	25 (57)	26 (52)	
None	43 (46)	19 (43)	24 (48)	
**Gallbladder stones (%)**				0.025
None	70 (74)	38 (86)	32 (64)	
Yes	24 (26)	6 (14)	18 (36)	
**Gallbladder polyp count (%)**				<0.001
Single	35 (37)	8 (18)	27 (54)	
Multiple	59 (63)	36 (82)	23 (46)	
**Time of polyp detection (%)**				0.401
<1 month	28 (30)	12 (27)	16 (32)	
1–12 months	44 (47)	19 (43)	25 (50)	
12–60 months	13 (14)	9 (20)	4 (8)	
>60 months	9 (10)	4 (9)	5 (10)	
**Polyp echo (%)**				0.276
Hypoechoic	8 (9)	2 (5)	6 (12)	
Weak echo	86 (91)	42 (95)	44 (88)	
**Abdominal symptoms (%)**				0.005
None	62 (66)	36 (82)	26 (52)	
Yes	32 (34)	8 (18)	24 (48)	
**Family history of gallbladder cancer (%)**				1
None	94 (100)	44 (100)	50 (100)	
Yes	0	0	0	
**Smoking history (%)**				0.468
None	93 (99)	43 (98)	50 (100)	
Yes	1 (1)	1 (2)	0 (0)	
**Alcohol consumption history (%)**				0.468
None	93 (99)	43 (98)	50 (100)	
Yes	1 (1)	1 (2)	0 (0)	
**Polyps base (%)**				< 0.001
Narrow base	51 (54)	34 (77)	17 (34)	
Wide base	43 (46)	10 (23)	33 (66)	
Age, *M* (*Q*1, *Q*3)	52.5 (39.25, 60)	52.5 (40, 62)	52.5 (38, 59.75)	0.924
Gallbladder wall thickness, *M* (*Q*1, *Q*3)	4 (3, 4)	4 (3.5, 4)	4 (3, 5)	0.253
Maximum polyp diameter, *M* (*Q*1, *Q*3)	12 (10, 17)	11 (8, 15)	14.5 (10, 18)	0.004
Polyps enlarged, *M* (*Q*1, *Q*3)	1 (0, 3)	1 (0, 3)	1.25 (0, 4)	0.499
CA_199, *M* (*Q*1, *Q*3)	8.11 (5.59, 15.07)	7.82 (5.64, 14.01)	9.41(4.38,17.72)	0.363
CEA, Mean (Quartile 1, Quartile 3)	1.55 (1.07, 2.1)	1.56 (1.02, 1.95)	1.54 (1.22, 2.47)	0.321

As shown in Table 3, polyp count, polyp base type, and polyp size were high-risk factors in the tumor group.

**Table 4.** Univariate analysis (training set).

Risk factors	Non-neoplastic group (*n* = 687)	Tumor group (*n* = 246)	*p* value
Cholecystitis (W–Cx)	3%	17%	<0.001
Solitary polyp (N–P)	33%	57%	<0.001
Hypoechoic (PE)	8%	19%	<0.001
Broad base (Bx)	27%	72%	<0.001
Age	49 years old	55.5 years	<0.001
Maximum diameter	11 mm	13 mm	<0.001

Comparing polyp feature indicators between the tumor and non-tumor groups in the training set revealed that the tumor group exhibited higher rates of concurrent cholecystitis, solitary polyps, hypoechoic lesions, and widened polyp bases. Additionally, older age and larger polyp size were more pronounced in the tumor group.

### 3.3. LASSO regression screening results on the training and validation sets

**Figure 1.**
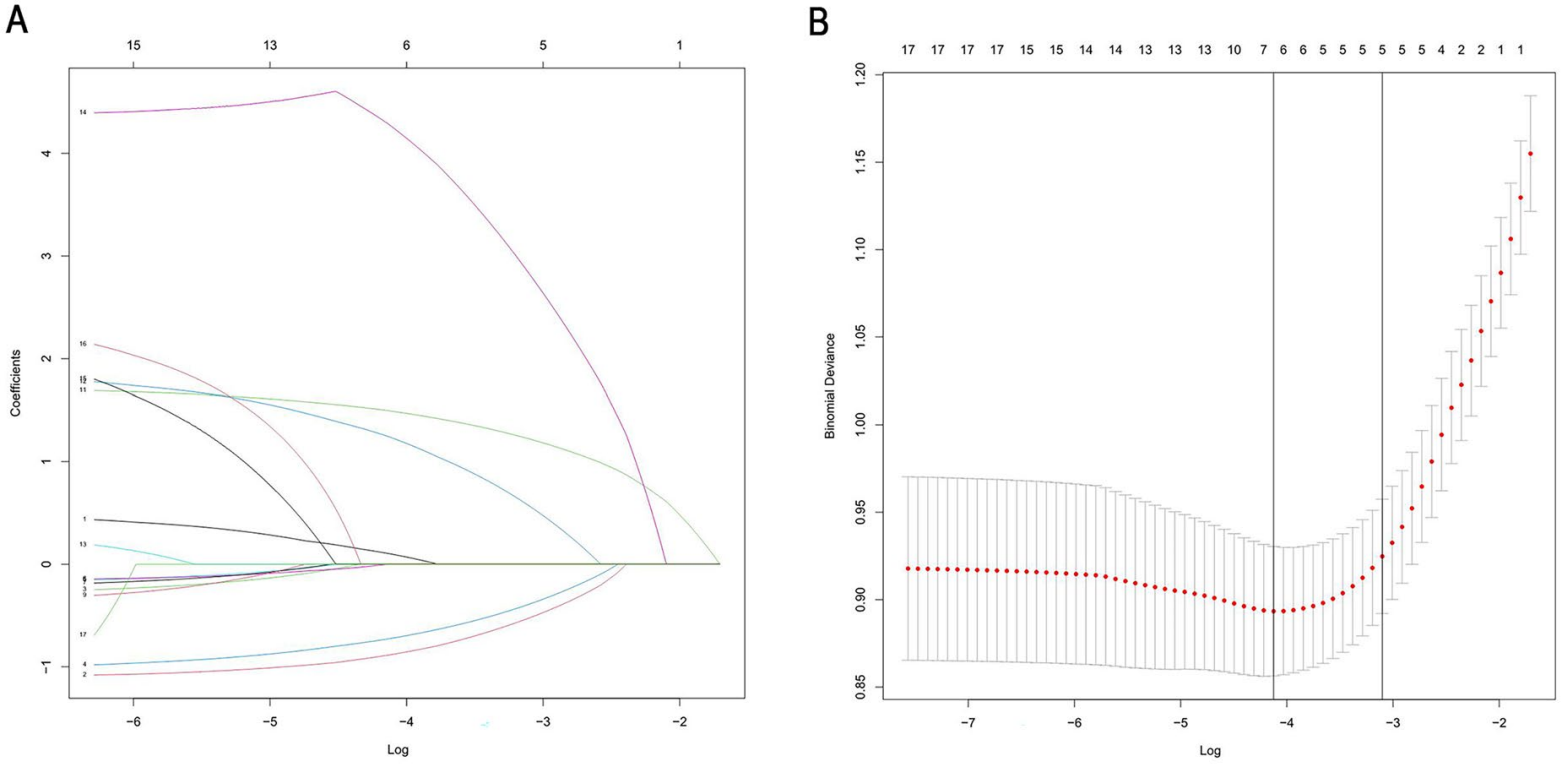
(A) Path diagram of LASSO coefficients for each variable; (B) cross-validation plot. Abbreviations: Abdominal symptoms: Abdo_Sx, Age: Age, Polyp Base: Bx, Carbohydrate Antigen 19: CA-199, Carcinoembryonic Antigen: CEA, Alcohol History: EHx, Polyp Formation Time: FTP, Gallbladder Wall Thickness: GWBT, History of Gallbladder Cancer: Hx_GB_Ca, Maximum Polyp Diameter: Max_P_Dia, Number of Polyps: N_P, Increased Polyp Diameter: P_Dia, Polyp Echo: PE, Smoking History: SHx, Sex: Sx, with Cholecystitis: W_Cx, with Gallstones: W_Gs.

All variables were included in the LASSO regression analysis. Through ten-fold cross-validation for iterative analysis, five variables closely associated with malignant transformation of GBP were identified: presence of concomitant cholecystitis, polyp count, polyp base width, age, and maximum polyp diameter. Figure 1A,B shows the LASSO coefficient path diagram and cross-validation plots.

### 3.4. Predictive model construction and validation

Based on the five variables selected *via* LASSO regression, an XGBoost machine learning model was constructed. The model demonstrated strong predictive ability on the training set, achieving an AUC of 0.862 (95% confidence interval [CI]: 0.8342–0.8893), accuracy of 83.3%, and sensitivity of 95.4%. Notably, its accuracy and sensitivity outperformed both the ESGAR guidelines (AUC = 0.71) and a previously reported Korean model (AUC = 0.79). This suggested strong generalization ability and clinical applicability. Consistent with this, the model also demonstrated superior performance in the validation set, further highlighting its effectiveness across different evaluation dimensions (Table 5).

**Table 5.** Performance evaluation of the XGBoost model in the training and validation sets.

Efficacy evaluation	Training set	Validation set
AUC (95% CI)	0.862 (0.8342–0.8893)	0.777 (0.6804–0.8737)
Accuracy (%)	83.3%	67.0%
Sensitivity (%) Specificity (%)	95.4% 49.6%	86.4% 50.0%

*Note*. AUC = area under the ROC curve; 95% CI = 95% Confidence interval; Accuracy: The proportion of correct predictions made by the model reflects its overall precision. Sensitivity (also known as recall): The model’s ability to correctly predict positive outcomes. Specificity: The model’s ability to correctly predict negative outcomes.

**Figure 2.**
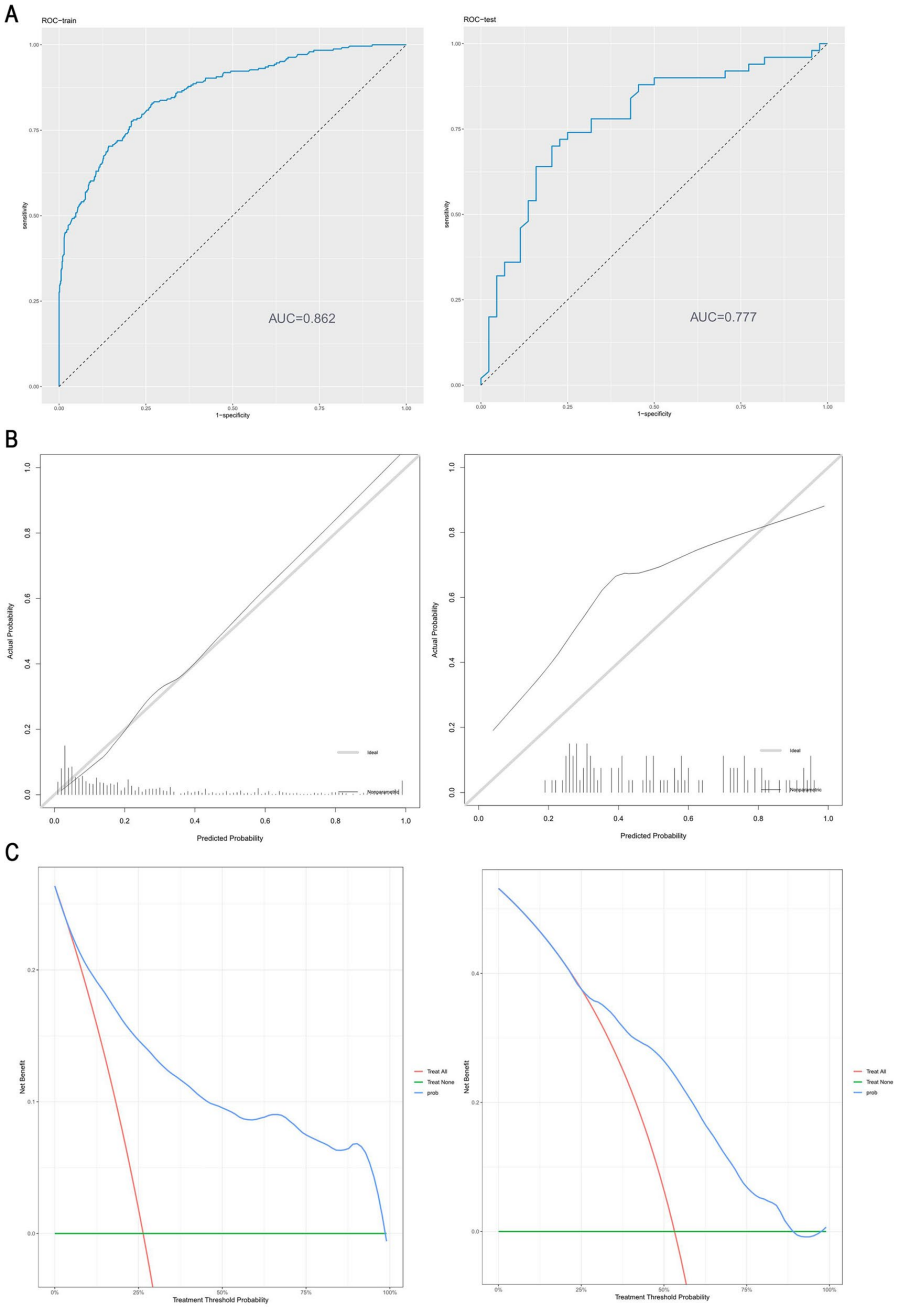
(A) ROC curve; (B) Calibration curve; (C) Decision curve.

The performance of this model was evaluated by its discriminatory ability and calibration (Figure 2A,B). Additionally, a decision curve was generated to quantify the net benefit of the model across various threshold probabilities, demonstrating its practical value for clinical decision-making (Figure 2C).

### 3.5. SHAP-based interpretability analysis

**Figure 3.**
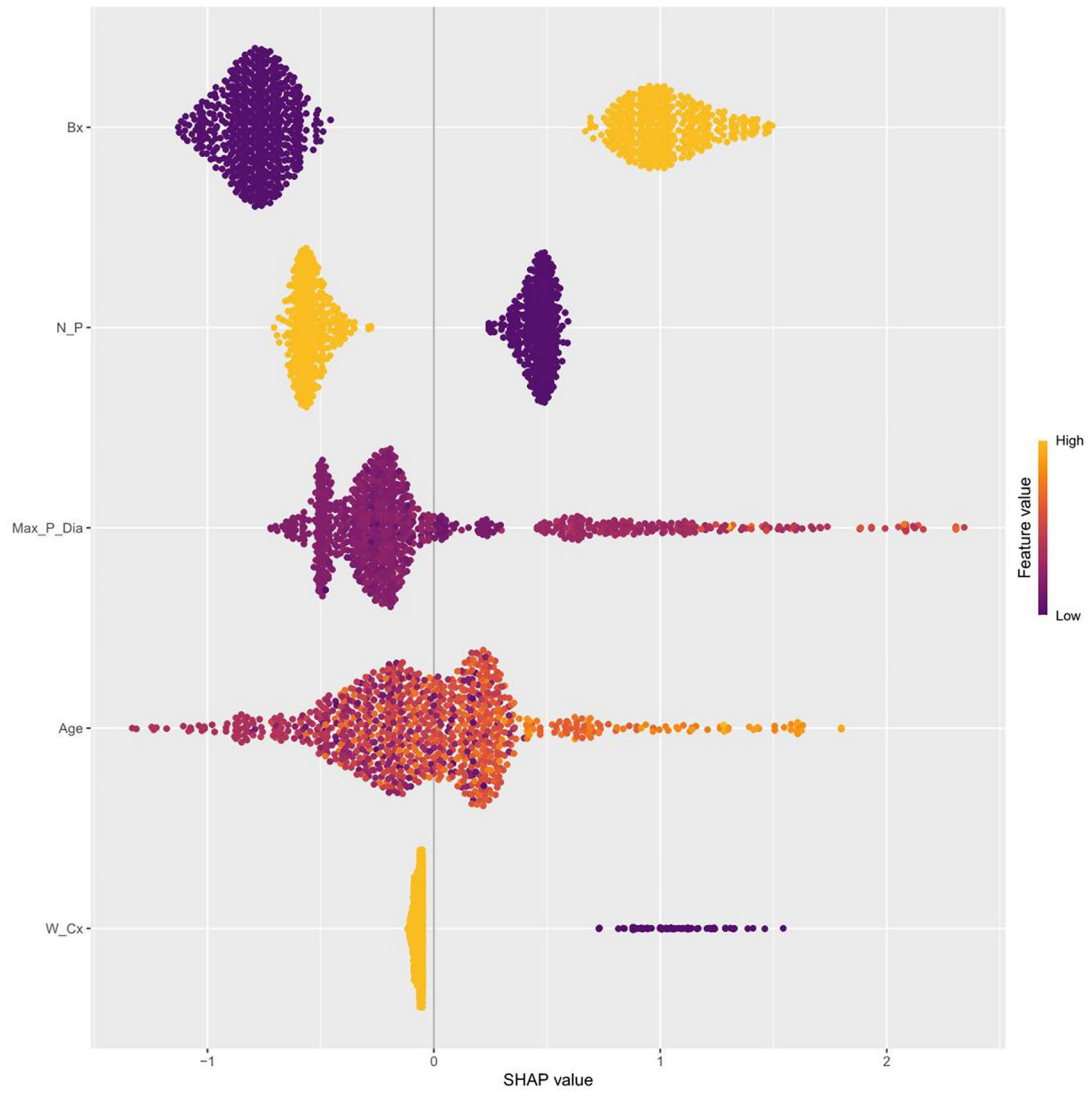
SHAP value plot. *Note*. The horizontal axis is labeled ‘SHAP values’, and the vertical axis ranks features based on their cumulative impact on predicted outcomes. This illustrates the hierarchy of importance of the different features. Each data point in the figure represents a specific sample instance. The position on the horizontal axis reflects the feature’s influence on the prediction. Positive numerical growth indicates a greater impact. Color maps the feature values of the cases. Yellow tones indicate high positive values, and purple tones indicate low negative values. This intuitively shows positive correlation. When data points overlap, they are displayed using vertical dispersion. This layout reflects the density distribution of SHAP values across samples, providing researchers with richer, more detailed visual information. It aids in deepening the understanding of how features influence the model across different sample scenarios.

Figure 3 presents the visualized SHAP values for each variable in the XGBoost model, along with the interaction results between variables and SHAP. The variables were ranked from highest to lowest contribution as follows: polyp base width, polyp count, maximum polyp diameter, age, and presence of concomitant cholecystitis. A higher absolute SHAP value indicated a greater risk of malignant transformation.

**Figure 4.**
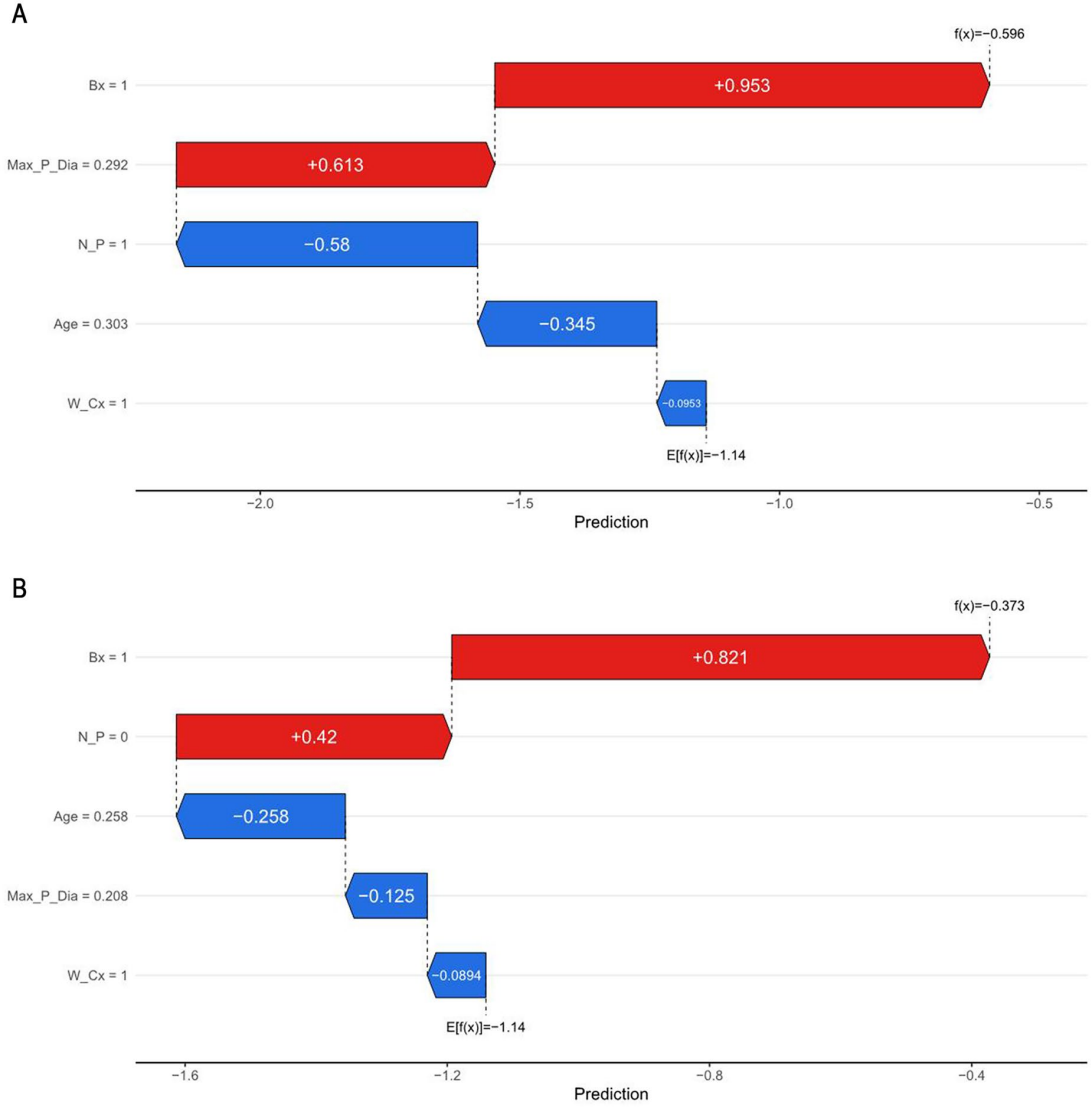
SHAP individual feature contribution plot for a case example.

Figure 4 focuses on a specific individual instance in the dataset and displays its feature contribution map. The length of each region on the map represents the contribution of each variable to the outcome. A longer region indicates a greater influence of that variable. Notably, the red region carries explicit significance, indicating that this variable promoted malignant transformation in GBP patients in this instance. The results revealed that age and polyp base type were the two risk factors most closely linked to neoplastic polyps. These factors accounted for 68% of the weight in this model and significantly increased the risk of neoplastic polyp transformation. Large solitary polyps (diameter: 13–14 mm) exhibited the highest risk in this model. This approach also laid the groundwork for future precision medicine research and clinical decision support.

## 4. Discussion

GBPs are a common clinical entity. Given their malignant potential and the poor prognosis of gallbladder cancer (5-year survival <5%) [11], most patients with GBPs are currently advised to undergo cholecystectomy. Clinical studies have shown that approximately 71.18% of polyps are non-neoplastic. However, neoplastic polyps are concerning, including gallbladder adenomas and gallbladder carcinoma. Adenomas are more prevalent. Studies have confirmed their significant malignant potential [12]. Consequently, researchers have proposed risk factors for predicting neoplastic transformation in GBPs [13–15], though clinicians find them challenging to apply due to their high operational complexity. This study aims to improve diagnostic efficiency by combining common clinical indicators with ultrasound findings.

The mechanism for malignant transformation of GBP remains inconclusive, although scholars generally propose two pathways: malignant transformation of adenomas and gallbladder mucosal metaplasia-dysplasia-malignant transformation. The latter pathway is likely more prevalent [16,17]. Multicentric loss of heterozygosity may correlate with the progression of gallbladder dysplasia to carcinoma, and mutations in K-Ras, p53, and p16 are critical steps in malignant transformation [18]. Progression from dysplasia to early-stage gallbladder carcinoma typically takes approximately 10 years. However, numerous reports indicate that gallbladder adenomas in polyp patients may become malignant. For instance, a Korean study reported a 23.5% probability of gallbladder adenoma transforming into gallbladder carcinoma [5]. Gallbladder adenomas are more prevalent than gallbladder carcinoma among polyps and often coexist with dysplasia. Therefore, their clinical characteristics warrant attention, especially since no sensitive and specific biomarkers for detecting gallbladder carcinoma are currently available clinically. As the disease progresses, many patients experience enlargement of gallbladder adenomas and subsequent malignant transformation [19]. This study identified five factors closely associated with neoplastic polyps: age, polyp diameter, polyp number, concomitant cholecystitis, and polyp base morphology. These factors were subsequently examined in detail. The analysis revealed that most cases of gallbladder adenomyomatosis involved cellular dysplasia and high-grade intraepithelial neoplasia. We then discussed the implications of these findings in relation to neoplastic polyps.

### 4.1. Age and malignant transformation

The risk of malignant transformation in GBPs increases with age. However, age thresholds vary across studies: some propose 50 years as the cutoff point for malignant transformation of polyps [6,20,21], while others suggest 60 years [22,23]. Other studies suggest that age is not a risk factor for malignant transformation [13]. In this study, patients over 55 years old exhibited a higher incidence of gallbladder adenoma than those aged 49, indicating that 55 years may represent a threshold at which the probability of malignant transformation significantly increases. Thus, age ≥55 years may serve as a predictive factor for gallbladder adenoma.

### 4.2. Polyp diameter and malignant transformation

The diameter of GBP is a significant factor in malignant transformation. However, existing literature shows considerable variations in the diameter threshold recommended for surgical intervention. Both Chinese and European guidelines recommend a surgical threshold of ≥10 mm for GBPs [6,24]. Park et al. [25] proposed that a threshold of >13 mm is more reasonable, reducing unnecessary cholecystectomies by 50% while minimizing missed diagnoses of gallbladder cancer. Other scholars contend that the risk of malignant transformation increases only when the polyp diameter exceeds 15 mm, warranting surgical intervention [26,27]. In our study, the mean diameter of adenomatous polyps (13 mm) was statistically significantly different from that of non-neoplastic polyps (11 mm). This observation suggests that polyps exceeding 13 mm are more prone to malignant transformation, a finding consistent with the report by Park et al.

### 4.3. Polyp number and malignant transformation

Our results confirm that solitary polyps are a risk factor for malignant transformation. Specifically, solitary polyps were more common in gallbladder adenomas, and adenomatous polyps represent an important pathway for malignant transformation of the gallbladder. These findings align with multiple reports suggesting that solitary polyps are more prone to malignant transformation [9,28,29]. Furthermore, this statistical analysis revealed that cholesterol polyps more frequently present as multiple polyps, thus corroborating that solitary polyps constitute a risk factor for malignant transformation.

### 4.4. Cholecystitis and malignant transformation

The European Multisociety Guidelines indicate that the coexistence of GBPs and cholecystitis may produce a synergistic effect, significantly increasing the risk of dysplasia and subsequent carcinogenesis in the gallbladder mucosa [6]. The pathological mechanism may involve the inflammatory environment of cholecystitis, promoting polyp growth. Inflammatory cells (e.g. macrophages) release various cytokines (e.g. TNF-α, IL-6) and growth factors, stimulating sustained proliferation of gallbladder epithelial cells and polyp cells. Increased cell division elevates the probability of DNA mutations. The abundant reactive oxygen species generated in the inflammatory environment cause oxidative DNA damage, accumulating oncogenic gene mutations (e.g. TP53, KRAS mutations). Additionally, inflammation disrupts normal tissue architecture and induces angiogenesis, providing nutritional support for the growth and potential malignant transformation of polyps [30]. Our findings support this perspective: the prevalence of concurrent gallbladder inflammation in gallbladder adenomas was 17%, significantly higher than the 3% observed in non-neoplastic polyps.

### 4.5. Polyp base morphology and malignant transformation

The wide-base morphology of GBPs has been identified as a risk factor for malignant transformation of the gallbladder [31,32]. While studies indicate that gallbladder adenomas can present as either narrow- or broad-based solitary polyps, clinical research shows that adenomas more commonly exhibit a broad-based morphology [33–35]. In this study, broad-based morphology was observed in 72% of neoplastic polyps, compared to 27% of non-neoplastic polyps, demonstrating a significant difference.

### 4.6. Gallbladder adenomyosis and malignant transformation

Gallbladder adenomyomatosis was previously considered a non-neoplastic polyp. However, updated research has revealed that it possesses the potential for malignant transformation [36,37]. Currently, numerous scholars worldwide regard gallbladder adenomyosis as a precancerous lesion with a malignant transformation rate ranging from 3% to 10%. This risk increases significantly when associated with gallbladder stones or when classified as the segmental type [38–41]. This study found that most adenomyomatomas were associated with cellular atypia or high-grade intraepithelial neoplasia, both of which are recognized as precancerous lesions. Therefore, this study classifies gallbladder adenomyomatoma as a neoplastic polyp.

### 4.7. Clinical interpretation and decision-making

In clinical practice, patients with broad-based polyps and low overall SHAP risk scores should undergo enhanced monitoring, with reassessment at least annually, rather than immediate surgery. Conversely, patients with multiple risk factors and high SHAP scores require surgical intervention.

This study has inherent limitations: 1. Our validation cohort had relatively few cases, potentially leading to model variability. Further extensive validation will stabilize the accuracy of the model before its clinical implementation. 2. The validation cohort exhibited high proportions of neoplastic polyps and concomitant cholecystitis. This high prevalence constitutes a clinically relevant ‘stress test’ for the model. Our model achieved an AUC of 0.777 and 86.4% sensitivity in external validation, robustly demonstrating its core predictive logic. Integrating multidimensional information—including polyp morphology (broad base), patient age, and concomitant cholecystitis—maintains stable discriminatory capability, even when applied to a real-world cohort with different distributions and ‘more concentrated’ disease severity.

## 5. Conclusion

This study established a multicenter, large-sample, preoperative prediction model for the malignant transformation of GBP. The constructed model is more effective than traditional tools that rely on polyp size alone for determining the need for surgery. Our analysis identified significant correlations between neoplastic polyps and age, polyp size, polyp number, cholecystitis, and polyp base morphology. This study provides valuable diagnostic references for distinguishing neoplastic polyps from non-neoplastic ones. The predictive model helps reduce unnecessary resection of non-neoplastic polyps and missed neoplastic polyps.

## Data Availability

The data that support the findings of this study are available from the corresponding author upon reasonable request.
